# Molecular Mechanisms of 2, 3′, 4, 4′, 5-Pentachlorobiphenyl-Induced Thyroid Dysfunction in FRTL-5 Cells

**DOI:** 10.1371/journal.pone.0120133

**Published:** 2015-03-19

**Authors:** Hui Yang, Huanhuan Chen, Hongwei Guo, Wen Li, Jinmei Tang, Bojin Xu, Minne Sun, Guoxian Ding, Lin Jiang, Dai Cui, Xuqin Zheng, Yu Duan

**Affiliations:** 1 Department of Endocrinology, First Affiliated Hospital of Nanjing Medical University, Nanjing, China; 2 Department of Gerontology, First Affiliated Hospital of Nanjing Medical University, Nanjing, China; Hokkaido University, JAPAN

## Abstract

Polychlorinated biphenyls (PCBs) can severely interfere with multiple animals and human systems. To explore the molecular mechanisms underlying 2, 3′, 4, 4′, 5- pentachlorobiphenyl (PCB118)-induced thyroid dysfunction, Fischer rat thyroid cell line-5(FRTL-5) cells were treated with either different concentrations of PCB118 or dimethyl sulfoxide (DMSO). The effects of PCB118 on FRTL-5 cells viability and apoptosis were assessed by using a Cell Counting Kit-8 assay and apoptosis assays, respectively. Quantitative real-time polymerase chain reaction was used to quantify protein kinase B (Akt), Forkhead box protein O3a (FoxO3a), and sodium/iodide symporter (NIS) mRNA expression levels. Western blotting was used to detect Akt, phospho-Akt (p-Akt), FoxO3a, phospho-FoxO3a (p-FoxO3a), and NIS protein levels. Luciferase reporter gene technology was used to detect the transcriptional activities of FoxO3a and NIS promoters. The effects of the constitutively active Akt (CA-Akt) and dominant-negative Akt (DN-Akt) plasmids on p-Akt, p-FoxO3a, and NIS levels were examined in PCB118-treated FRTL-5 cells. The effects of FoxO3a siRNA on FoxO3a, p-FoxO3a, and NIS protein levels were examined in the PCB118-treated FRTL-5 cells. The effects of pcDNA3 (plsmid vectors designed for high-level stable and transient expression in mammalian host)-FoxO3a on NIS promoter activity were examined in the PCB118-treated FRTL-5 cells. Our results indicated that relatively higher PCB118 concentrations can inhibit cell viability in a concentration- and time-dependent manner. Akt, p-Akt, and p-FoxO3a protein or mRNA levels increased significantly in PCB118-treated groups and NIS protein and mRNA levels decreased considerably compared with the control groups. FoxO3a promoter activity increased significantly, whereas NIS promoter activity decreased. These effects on p-FoxO3a and NIS could be decreased by the DN-Akt plasmid, enhanced by the CA-Akt plasmid, and blocked by FoxO3a siRNA. The overexpressed FoxO3a could reduce NIS promoter activity. Our results suggested that PCB118 induces thyroid cell dysfunction through the Akt/FoxO3a/NIS signaling pathway.

## Introduction

Polychlorinated biphenyls (PCBs) are a type of typical environmental endocrine disruptors present in persistent organic pollutants. Because of their stable chemical properties and perfect insulativity, they were widely used in many industries as flame retardants, transformers, capacitors, lubricants, and plasticizers until their production was banned in 1979. However, owing to their lipophilic and biological persistence, significant concentrations of PCBs still exist in the environment. Because they can easily accumulate in adipose tissue, they gradually accumulate up the food chain, and affect human health by damaging multiple organs. Numerous research studies have indicated that long-term exposure to PCBs produces adverse effects on the immune, reproductive, and nervous systems. PCBs have also been associated with hepatotoxicity and lipotoxicity, especially in newborns, young children, and adolescents [1–4]. In addition, PCBs are thought to affect thyroid function. When harbor seals and Beluga whales were exposed to various organochlorine compounds, mainly PCBs, a high incidence of goiter was observed [5]. It was declared that PCBs could interfere with thyroid hormone synthesis, secretion, transportation, and metabolism. Our early findings suggested that low concentrations of 2,3′,4,4′,5-pentachlorobiphenyl or continuous exposure in animal models to polychlorinated biphenyl 118 (PCB118) induced abnormal thyroid morphology, dramatically decreasing the expression of thyroidal sodium/iodide symporter [NIS or solute carrier family 5, member 5 (SLC5A5)] at the transcriptional level [6]; however, the underlying mechanism was unknown [7–9].

**Fig 1:**
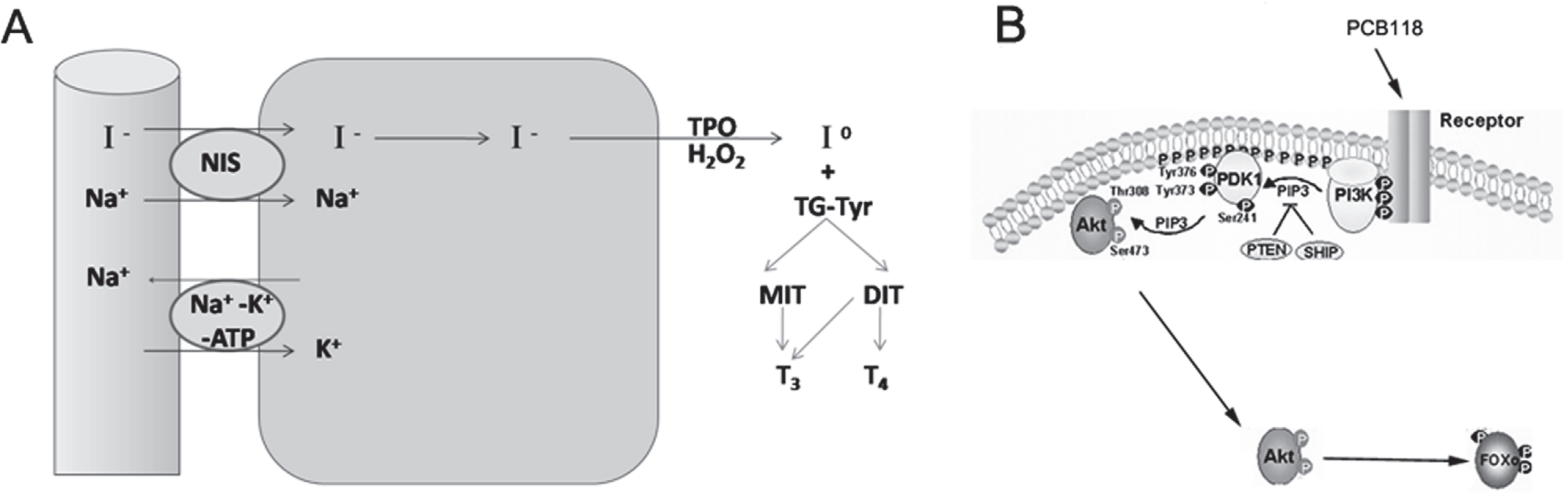
Functional diagrams of NIS and the PI3K/Akt/FoxO3a pathway. NIS is located on the outer membrane of the thyrocyte. Its function is to pump sodium out of the follicular cells while pumping iodide into the follicular cells (A); PCB118 stimulates cells, activates the PI3K/Akt signaling pathway, and increases Akt phosphorylation levels, after which p-Akt phosphorylates its downstream target gene FoxO3a (B).

NIS is located on the outer membrane of thyrocytes. Its function is to pump sodium out of follicular cells while pumping iodide into the cells, thus constituting the first step in the biosynthesis of iodine-containing hormones such as triiodothyronine and thyroxine [10] (Fig. 1A). NIS activity is sensitive to both iodine availability and thyroid-stimulating hormone (TSH) stimulation, and without it, iodine would not be imported into the follicular cells in sufficiently high concentrations to produce adequate amounts of thyroxine [11]. Numerous studies have indicated that cyclic adenosine monophosphate, protein kinase A, and protein kinase C are involved in mediating NIS transcription activity [10], but Kogai et al. also reported that decreased NIS mRNA expression and NIS trafficking to the plasma membrane were found in differentiated thyroid cancers, which may be attributed to the activation of the insulin/phosphoinositide-3-kinase (PI3K)/Akt signaling pathway [11]. In addition, the activated PI3K/Akt pathway inhibits NIS expression and function in thyrocytes [12]. Akt (or protein kinase B), a serine/threonine kinase, is one of the primary kinases activated by PI3K and is a central regulator of cellular processes, including proliferation, differentiation, migration, survival, metabolism, and therapeutic resistance [13–17]. On the other hand, Forkhead box protein O3a (FoxO3a), which belongs to Forkhead transcription factors of the FoxO subfamily (FoxOs), is one of the most important downstream targets of the PI3K/Akt pathway [18], which is reportedly involved in cell cycle arrest [19], apoptosis [20], and the oxidative stress response [21,22]. FoxO3a localizes in the nucleus, where it activates the transcription of target genes and is phosphorylated by activated Akt; phosphorylated FoxO3a transfers to the cytoplasm where its transcriptional activity is inhibited [23] (Fig. 1B).Although it has been confirmed that several transcription factors regulate NIS expression by binding to a NIS promoter, such as β-catenin, Pax8, p53 [24,25], it has not been confirmed whether FoxO3a plays a role in NIS expression.

Fischer rat thyroid cell line-5 (FRTL-5) is a functional clone of cells that behave in a manner similar to normal thyrocytes in vitro and has been used as a model for studying thyroid cell functions relevant to human pathophysiology. It is the most preferred and frequently used thyroid cell line for its simplicity, accessibility, and ability of allowing permanent transfections [26]. Previous studies showed that treatment with the non-steroidal anti-inflammatory drug sulindac sulfide reverses the PI3K/Akt-mediated cytoplasmic accumulation of FoxO3a and restores its transcriptional activity in FRTL-5 cells [27]; The aforementioned results indicated that the activity of FRTL-5 cells was involved in the PI3K/Akt/FoxO3a pathway.

Here, we proposed that PCBs may induce abnormal NIS expression by activating the PI3K/Akt pathway and result in thyroid dysfunction. We selected PCB118 to investigate the molecular mechanisms underlying PCB-induced thyroid dysfunction in FRTL-5 rat cells in our study.

## Materials and Methods

### Materials

FRTL-5 cells were kindly provided by Dr. Zheng Xuqin (Department of Endocrinology, First Affiliated Hospital of Nanjing Medical University) [28], who was kindly gifted these cells by Professor Michael Derwahl (Department of Medicine, St. Hedwig Hospital) [29]. Coon’s modified Ham’s F-12 medium, transferrin, bovine insulin, hydrocortisone, somatostatin, glycyl-L-histidyl-L-lysine acetate, and bovine TSH were purchased from Sigma-Aldrich (USA). Newborn calf serum was purchased from Gibco (USA). Penicillin and streptomycin were purchased from HyClone (USA). PCB118 (purity: 100%, analyzed using GC/MS) was purchased from AccuStandard (USA) and was dissolved initially in 612 μL dimethyl sulfoxide (DMSO) and maintained as a 25 mM stock solution in the dark. When needed, this solution was diluted to the required concentration with culture media. The final concentration of DMSO in culture medium was maintained below 0.1%.

### Cell culture

FRTL-5 cells were maintained in Coon’s modified Ham’s F-12 medium supplemented with 5% newborn calf serum, 100-U/mL penicillin,100-μg/mL streptomycin and a six-hormone mixture containing 10-μg/mL bovine insulin, 0.36-ng/mL hydrocortisone, 5-μg/mL transferrin, 2-ng/mL glycyl-L-histidyl-L-lysine acetate, 10-ng/mL somatostatin and 1-mU/mL bovine TSH. The cells were maintained in 5% CO_2_ at 37°C and passaged every 7–10 days as described previously [30]. We observed cells under a microscope, and the experiments were initiated when the proportion of adherent cells on the culture plate approached 80%. When seeding cells, we digested and counted cells to ensure that an equal number of cells were seeded into each well.

### Cell viability assay

The Cell-Counting Kit-8 (CCK-8; Beyotime, China) viability assay was used as an index of cell viability according to the manufacturer’s recommendations. In brief, FRTL-5 cells were seeded in 96-well plates (100μL, 1 × 10^4^cells/well) and cultured overnight. Cells were divided into the following three groups: (1) blank control (BC) group, in which cells were incubated with culture medium without DMSO or PCB118; (2) DMSO (solvent) control group, in which cells were incubated with culture medium and DMSO; and (3) PCB118 treated group, in which cells were incubated with culture medium and PCB118; the latter was further subdivided according to the final PCB118 concentrations of 0.025, 0.25, 2.5, 25, 250, 2500, and 25000 nM, respectively. Cells were separately incubated for 24, 48, or 72 h. Subsequently, 10μL of CCK8 was added to each well, and the cells were incubated for a further 2–3 h. Under the action of mitochondrial dehydrogenase in cells, CCK8 could be reduced to a yellow carapace product with high water solubility named as formazan, and the amount of formazan produced is positively correlated with cell viability. The absorbance was determined at 450 nm using a microplate reader, and the absorbance value indirectly reflected the number of viable cells. We then measured the absorbance values of the reaction product formazan at 450 nm for each well using a microplate reader. The relative level of cell viability in each group of cells was calculated according to a previously described formula: P = A1/A2×100% in which P was relatively cell viability ratio; A1 was mean absorbance value of PCB118-treated cells; and A2 was mean absorbance value of solvent control cells [31]. All determinations were performed in quintuplicate.

### Cell apoptosis assay

On the basis of the results of the cell viability assay, the PCB118-treated groups with concentrations of 0.025–25 nM were selected. At these concentrations, cell viability did not decrease significantly compared with the control groups. Then, we seeded the FRTL-5 cells in 6-well plates (2 mL, 8 × 10^4^ cells/well), cultured the plates overnight, and stimulated the cells with low PCB118 concentrations (0.025–25 nM). We observed the 6-well plates under a microscope and found that apoptosis began to appear approximately 72 h after PCB118 treatment; thus, we selected 72 h as the time point to perform the cell apoptosis assay. The cells were harvested 72h after PCB118 treatment and double-stained with annexin V-fluorescein isothiocyanate (FITC) and propidium iodide according to the manufacturer’s protocol. Cell samples were quantitatively analyzed on a FACSVantage SE flow cytometer (BD Biosciences, USA) and apoptotic fractions were determined. Annexin V–FITC-positive cells reflected the relative proportion of apoptotic cells.

### Quantitative real-time polymerase chain reaction (qRT-PCR) analysis of Akt, FoxO3a, and NIS

FRTL-5 cells were seeded in 6-well plates (2 mL, 16 × 10^4^ cells/well), cultured overnight, and treated with low PCB118 concentrations (0.025–25 nM) for 24 h. At these concentrations, cells viability was not significantly decreased and showed no apoptotic difference compared with the control groups. Total RNA was isolated using RNAiso Plus (Takara, Japan) following the manufacturer’s instructions and reverse transcribed to complementary DNAs using the PrimeScript RT Master Mix Kit (Takara). Quantitative RT-PCR was performed following a standard SYBR-Green PCR kit protocol on a Step One Plus system (Applied Biosystems, USA). β-actin was used as an endogenous control to normalize the total amount of mRNA in each sample. Relative expression levels of target genes were calculated using the 2 ^−ΔΔCT^ method [32]. The following primers were used: rat Akt, FoxO3a, NIS, and β-actin (all primers were synthesized by Takara); the primers are presented in Table 1.

**Table 1:** Primers used for quantitative real-time PCR.

Primer	Primer sequences (5′ to 3′)	Genbank
Akt	Forward: GCCCAACACCTTCATCATCC	NM-033230.2
	Reverse: GTCTCCTCCTCCTGCCGTTT	
FoxO3a	Forward: TGCTAAGCAGGCCTCATCTCAA	NM-001106395
	Reverse: AGATGGCGTGGGAGTCACAA	
NIS	Forward: CCAAGAAGGCCAATCACA	NM-052983.2
	Reverse: CCGCTGCCTACTGAAATCT	
β—actin	Forward: GGAGATTACTGCCCTGGCTCCTA	NM-031144.3
	Reverse: GACTCATCGTACTCCTGCTTGCTG	

### Western blotting analysis of Akt, p-Akt, FoxO3a, p-FoxO3a, and NIS

FRTL-5 cells were seeded in 6-well plates (2 mL, 15 × 10^4^ cells/well) and treated as described above. After being cultured for 48 h with PCB118 or DMSO, proteins were extracted using a protein extraction kit (KeyGEN Biotech, China) according to the manufacturer’s directions. Protein concentrations of the samples were measured using the Bio-Rad protein assay (Bio-Rad, USA). Then, 30μg of protein was denatured with 2% sodium dodecyl sulfate (SDS) and 50 mM dithiothreitol, loaded on an SDS-PAGE gel (10%), and transferred to a polyvinylidene fluoride membrane (Millipore, USA) using a standard protocol. Western blotting was performed as described previously [33], except for protein visualization, which was performed using an enhanced chemiluminescence reagent (Thermo, USA). The antibodies used in this procedure were Akt (1:1000 dilution), p-Akt (1:2000 dilution), FoxO3a (1:1000 dilution), glyceraldehyde 3-phosphate dehydrogenase (GAPDH, 1:1000 dilution), all purchased from Cell Signaling Technology (USA). p-FoxO3a(1:1000 dilution) was purchased from Abcam (USA), and NIS (1:250 dilution) was purchased from Bioss (USA), all primary antibodies were generated in rabbits. The secondary antibodies (goat anti-rabbit, 1:4000 dilution) were obtained from Bioworld (USA). GAPDH was used as the endogenous control to adjust the variation caused by protein loading.

### Activation or suppression for the PI3K/Akt signaling pathway

The constitutively active Akt (CA-Akt) and dominant-negative Akt (DN-Akt) plasmids were constructed by Sunbio (Shanghai, China). FRTL-5 cells were seeded in 6-well plates to approximately 50% confluence and washed with 2 mL of warmed serum-free culture medium. Then, 2 mL of a premade plasmid/Lipofectamine mixture were added. This mixture was prepared by incubating 4μg of CA-Akt plasmid (DN-Akt or BC plasmid) with 10μL of Lipofectamine 2000 Transfection Reagent (Invitrogen, USA) and serum-free medium for 20 min at room temperature. The cells were randomly divided into three groups. The BC, CA-Akt, and DN-Akt groups consisted of cells treated with the BC, CA-Akt, and DN-Akt plasmids, respectively. The cells were incubated for 6 h before being refreshed with 2 mL of complete medium. Incubation of cells continued for 24 h, and then PCB118 (final concentration, 25 nM) or DMSO was added. After the cells were treated with PCB118 for 24 h, proteins were extracted and western blotting was performed to observe the effects of CA-Akt and DN-Akt on p-Akt, p-FoxO3a, and NIS levels in the FRTL-5 cells.

### Plasmid construction of FoxO3a promoter and NIS promoter

The NIS and FoxO3a gene sequences were searched using the NCBI Genome Database. Promoter prediction was conducted using McPromoter software, and then, the promoter sequences were verified using the dual luciferase reporter system. Finally, we determined the genomic sequences of the NIS promoter (5’-2235 to 5’-50 bp) and FoxO3a promoter (5’-2000 to 5’-20 bp). NIS promoter and FoxO3a promoter were amplified by PCR using the following primers: FoxO3a-forward: CCGAGCTCTTACGCGTTGTATTTACTCGTAAATGGGCC; FoxO3a-reverse: GATCGCAGATCTCGAGAGGGACGAGGCGGGAGG; NIS-forward: CCGAGCTCTTACGCGTCAACCCCACTGCAGTTTGTG; and NIS-reverse: GATCGCAGATCTCGAGGGAGACAGGTGACTCGGTGAG. The PCR product was subcloned into the luciferase reporter gene plasmid pGL3-basic (Invitrogen, USA) and termed FoxO3a-promoter-Luc or NIS-promoter-Luc. Wide-type pcDNA3-FoxO3a and BC plasmid were constructed by Invitrogen (USA). All inserts were confirmed by DNA sequencing.

### Luciferase reporter assays of FoxO3a and NIS promoters

X-tremeGENE HP DNA Transfection Reagent (Roche, USA) was used to transfect a promoter/reporter gene into FRTL-5 cells. In brief, the cells were grown in 24-well plates to approximately 50% confluence and refreshed with 500μL of warmed (37°C) serum-free culture medium. Then, 50μL of a premade plasmid/transfection reagent mixture was added. This mixture contained serum-free culture medium, X-tremeGENE HP DNA Transfection Reagent, promoter-Luc plasmid, and renilla plasmid (the volume ratio of promoter plasmid and transfection reagent was 1:2, and the mass ratio of the promoter and renilla plasmids was 1:1000). PCB118 was added at different concentrations (0.025–25 nM) 24 h after transfection, and reporter activity was measured after 48 h. To determine the influence of FoxO3a on the NIS promoter, 0.15μg pcDNA3-FoxO3a or BC plasmid was co-transfected with 0.15μg NIS promoter plasmid. PCB118 at 25 nM or DMSO was added 24 h after transfection, and reporter activity was measured after 48 h. To measure luciferase activity, the cells were washed twice with 1 mL of 1× PBS, lysed with 100μL of 1× passive lysis buffer (Promega, USA), shaken for 15 min at room temperature, and pipetted repetitively for 30 times using a yellow-tip micropipette (200μL capacity). The lysate was then centrifuged at 12,000 rpm for 5 min at room temperature. Luciferase activity was analyzed using a Dual-Luciferase Reporter Assay System (Promega) according to the manufacturer’s protocol using a Glomax Luminometer (Promega). Data represented the mean ± standard deviation (SD) of triplicate samples and were expressed as relative values.

### FoxO3a-specific small interfering RNA(siRNA)analysis

To transiently knockdown the expression of the genes of interest, we used FoxO3a-targeting siRNA. FRTL-5 cells were seeded in 6-well plates to approximately 50% confluence and washed with 2 mL of warmed serum-free culture medium. Then, 2 mL of a premade siRNA/Lipofectamine mixture was added. This mixture was prepared by incubating 5μL of siRNA with 5μL of Lipofectamine 2000 Transfection Reagent (Invitrogen) and 500μL of serum-free medium for 20 min at room temperature, and then diluting it with 1.5 mL of serum-free medium. The cells were randomly divided into two groups. The negative control (NC) group consisted of cells treated with non-target siRNA. The FoxO3a-siRNA group consisted of cells treated with FoxO3a siRNA (target siRNA). The cells were incubated for 6 h before being placed in serum-free medium with 2 mL of complete medium. The cells were incubated for 24 h, and then PCB118 (final concentration, 25 nM) or DMSO was added. After the cells were treated with PCB118 or DMSO for 48 h, proteins were extracted and western blotting was performed to observe the effects of siRNA on FoxO3a, p-FoxO3a, and NIS levels in the FRTL-5 cells. Both FoxO3a and NC siRNAs were synthesized by Invitrogen (USA).

### Statistical analysis

Data are presented as the mean ± SD of at least three independent experiments. The SPSS 13.0 statistical package (SPSS Inc., Chicago, IL, USA) was used to perform paired *t*-tests and one-way analysis of variance (ANOVA), the least significant difference (LSD) was used to perform post-hoc analysis after ANOVA, and a p value of <0.05 was considered significant.

## Results

### PCB118 inhibited cell viability in a concentration and time-dependent manners

**Fig 2:**
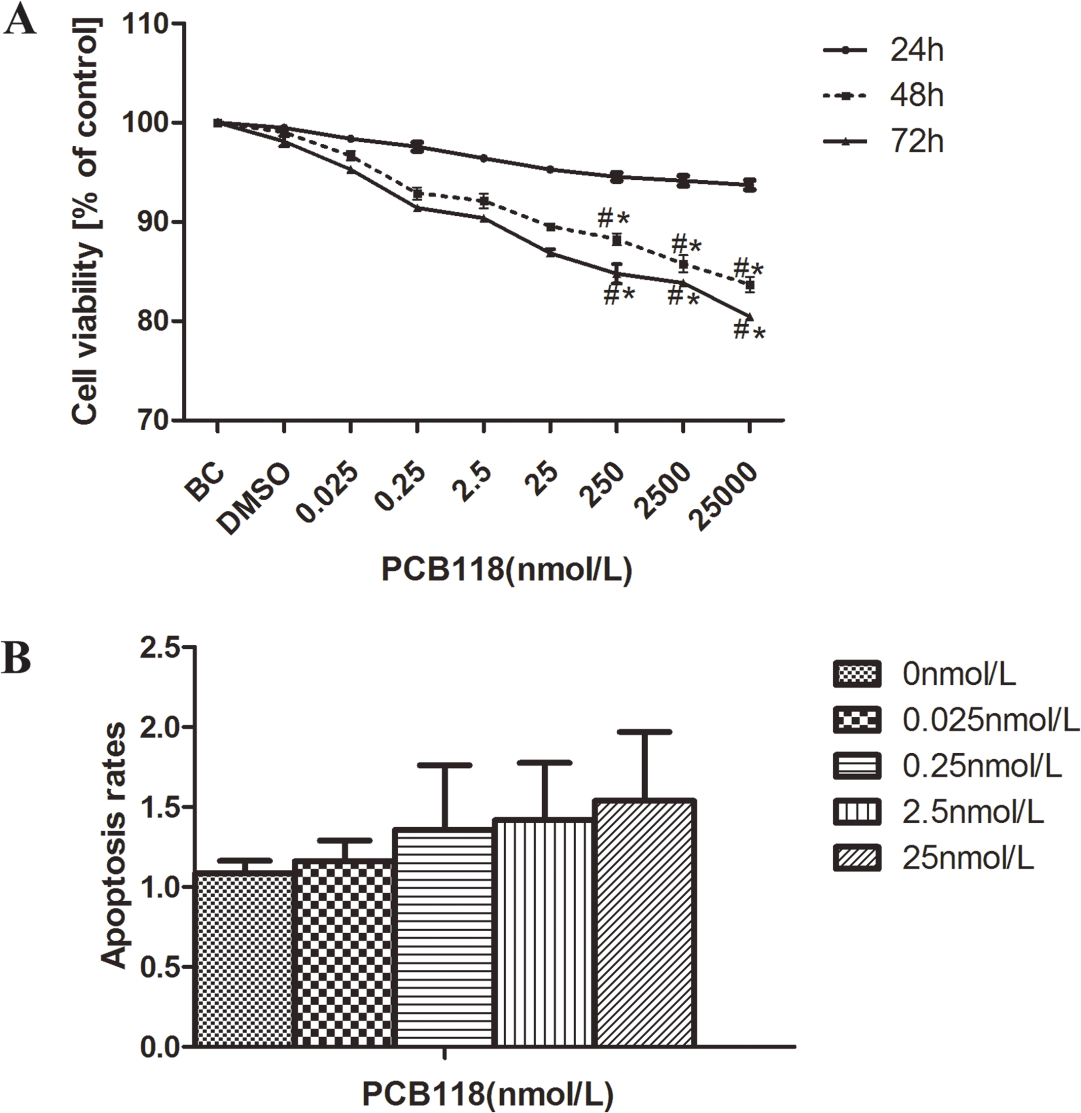
Effect of PCB118 on FRTL-5 cell viability and apoptosis. Cells were treated with PCB118 at different concentrations (0.025–25000 nM in the Cell Counting Kit-8 (CCK8) assay and 0.025–25 nM in the apoptosis assay). The viability was measured using the CCK-8 assay (A). The apoptosis rate was measured using a FACSVantage SE flow cytometer (B). Data are presented as the mean ± SD of three independent experiments.*p < 0.05, compared with the DMSO control group. ^#^p < 0.05, compared with the low PCB118-treated groups (0.025, 0.25, 2.5, and 25 nM).

As shown in Fig. 2A, cells were stimulated with media culture, DMSO, or different concentrations of PCB118 (0.025–25000 nM) for 24h, 48h, and 72 h. From 24 h to 72 h, no significant difference was observed between the BC and DMSO groups (p > 0.05). Within 24 h, no evident difference in cell viability was observed between PCB118 (0.025–25000 nM)-treated groups and the DMSO control group (p > 0.05), and no significant difference was observed between each PCB118-treated group (p > 0.05). However, relatively higher PCB118 concentrations (250–25000 nM) could significantly decrease cell viability compared with the DMSO control group or low PCB118 concentrations (0.025, 0.25, 2.5, 25nM)-treated groups (p < 0.05) when the incubation time was extended to 48 and 72 h. Thus, PCB118 could suppress cell viability in a concentration- and time-dependent manner.

Therefore, low PCB118 concentrations (0.025, 0.25, 2.5, and 25 nM) were selected in cell apoptosis experiment in FRTL-5 cells.

### Low PCB118 concentrations did not induce cell apoptosis

The cell apoptosis assay was performed to determine whether relatively low PCB118 concentrations (0.025–25 nM) could induce cell apoptosis. As shown in Fig. 2B, no significant difference was observed in the cell apoptosis rates of PCB118-treated and DMSO control groups (p > 0.05), and the difference between each PCB118-treated group was also not significant (p > 0.05).

Because our results indicated that PCB118 at high concentrations (250–25000 nM) is an effective inhibitor of FRTL-5 cell viability and PCB118 at low concentrations (0.025–25 nM) does not induce cell apoptosis or influence cell viability, we selected PCB118 concentrations of 0.025–25 nM for subsequent experiments.

### PCB118 treatment decreases NIS expression

**Fig 3:**
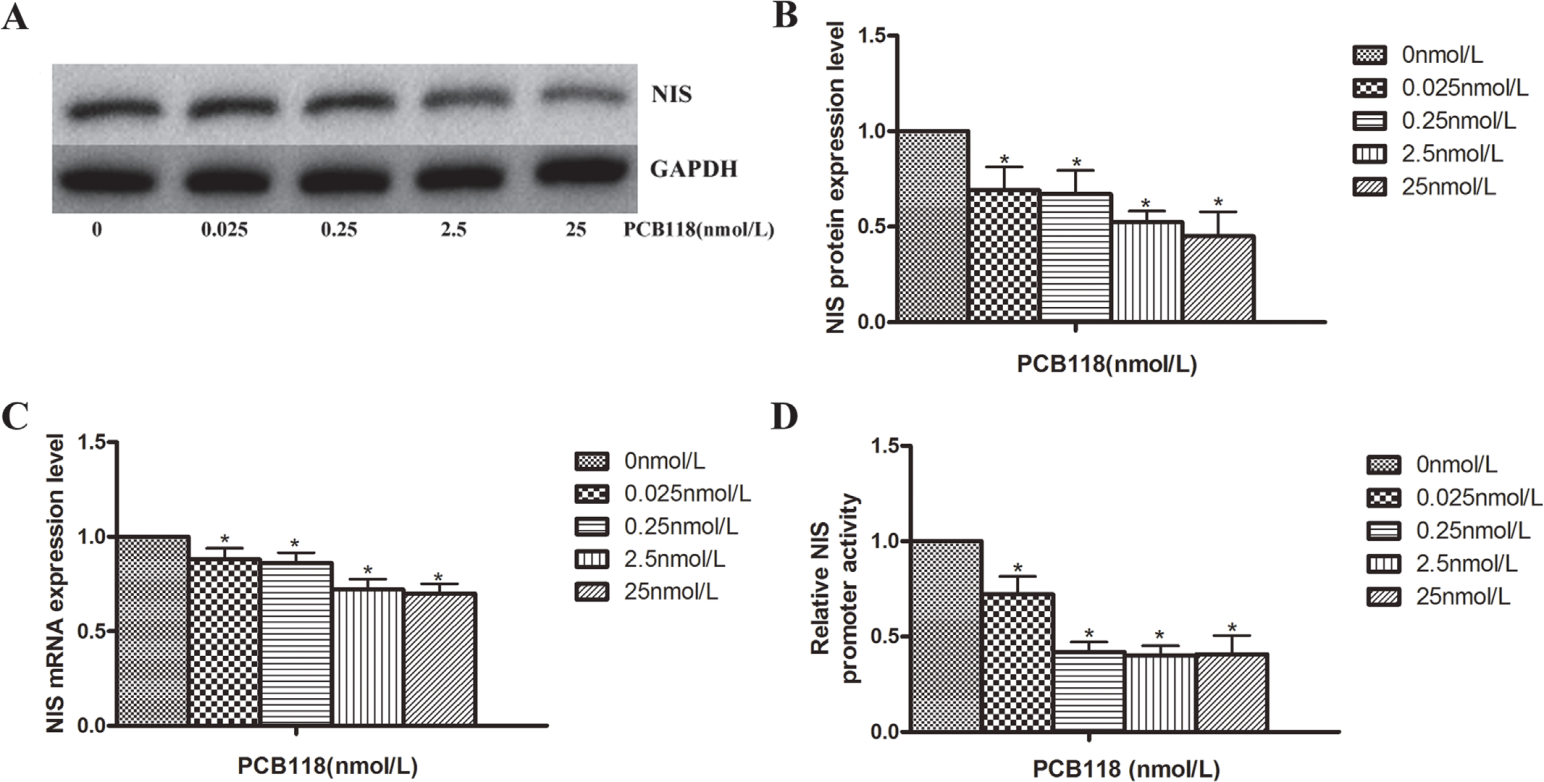
Exposure of FRTL-5 cells to PCB118 results in the down-regulation of NIS expression. Cells were treated with DMSO or various concentrations of PCB118 (0.025–25 nM) for 24 or 48 h. Whole cell lysates were analyzed by western blotting using antibodies recognizing NIS (A and B). Total RNA was isolated for qRT-PCR of NIS (C), and the FRTL-5 cells were harvested for the measurement of NIS promoter activity (D). Results show sections of blots from one experiment of the three that yielded similar results (A) or represented the mean ± SD of three independent experiments (B, C, and D). GAPDH served as the loading control in western blotting.*p < 0.05, compared with the DMSO control group. The relative ratio of target mRNA/β-actin, target protein/GAPDH and promoter activity in solvent control group was set as 1.

To investigate whether PCB118 had an effect on NIS expression, western blotting, qRT-PCR, and luciferase reporter assays of the NIS promoter were performed. As shown in Fig. 3, after treatment with various concentrations of PCB118 for 24 h (for qRT-PCR analysis) or 48 h (for Western blot analysis and luciferase reporter assays), both protein (Fig. 3A, B) and mRNA levels (Fig. 3C) of NIS decreased significantly (p < 0.05) after treatment with low PCB118 concentrations (0.025–25 nM) compared with the DMSO control group. Similar to the mRNA and protein results, PCB118 suppressed NIS promoter activity significantly (p < 0.05; Fig. 3D). However, no significant difference was observed between each PCB118-treated group (p > 0.05). Altogether, these results suggest that PCB118 down-regulates NIS expression at both the mRNA and protein levels. Furthermore, such results were observed occurred at the transcriptional level.

### FoxO3a down-regulates NIS expression

**Fig 4:**
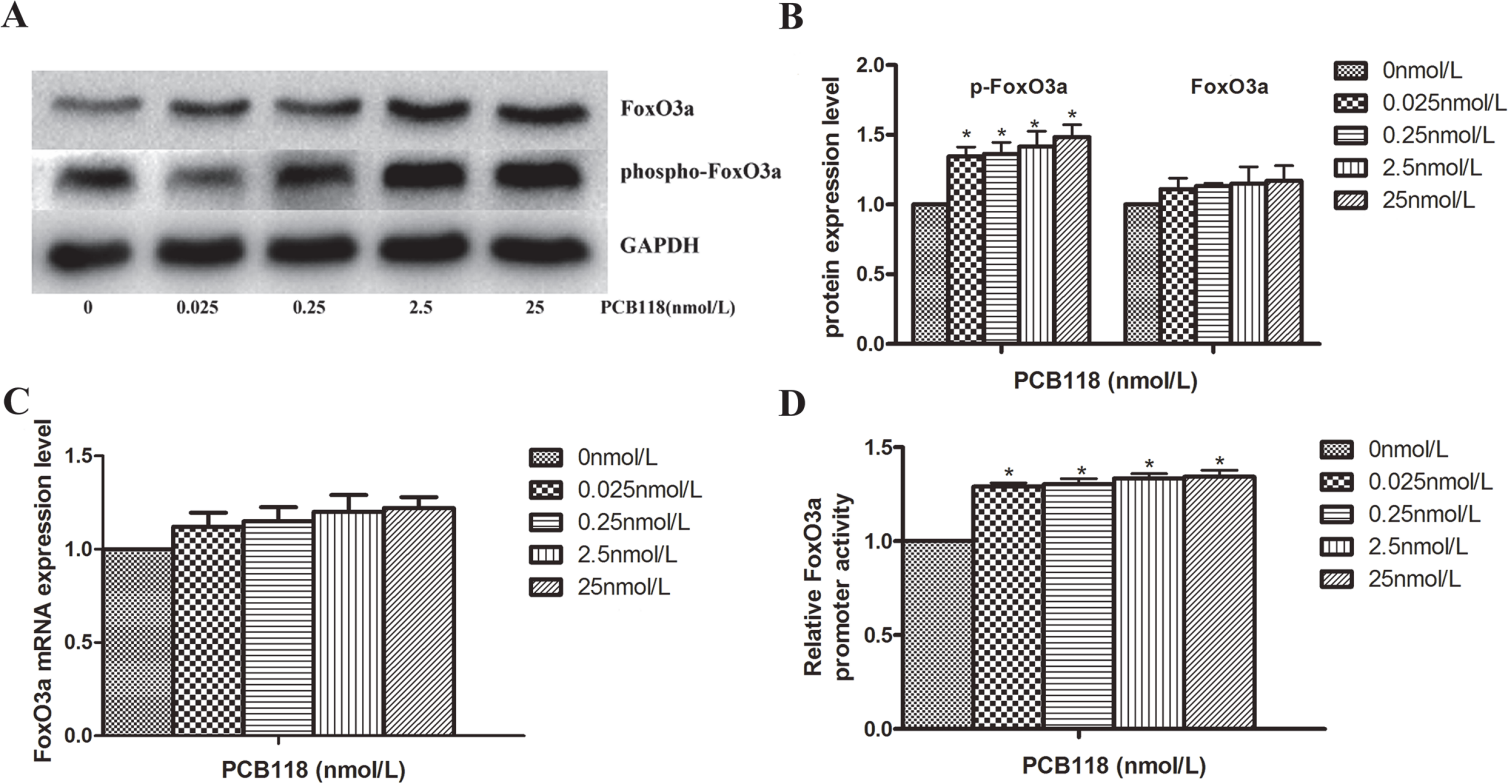
Exposure of FRTL-5 cells to PCB118 results in the up-regulation of FoxO3a or p-FoxO3a expression. Cells were treated with DMSO or various concentrations of PCB118 (0.025–25 nM) for 24 or 48 h. Whole cell lysates were analyzed by western blotting using antibodies recognizing FoxO3a and p-FoxO3a. GAPDH served as the loading control (A and B). Total RNA was isolated for qRT-PCR of FoxO3a (C), and the PCB118-treated FRTL-5 cells were harvested for the measurement of FoxO3a promoter activity (D). Results show sections of blots from one experiment of the three that yielded similar results (A) or represent the mean ± SD of three independent experiments (B, C, and D). GAPDH served as the loading control in western blotting.*p < 0.05, compared with the DMSO control group. The relative ratio of target mRNA/β-actin, target protein/GAPDH and promoter activity in solvent control group was set as 1.

**Fig 5:**
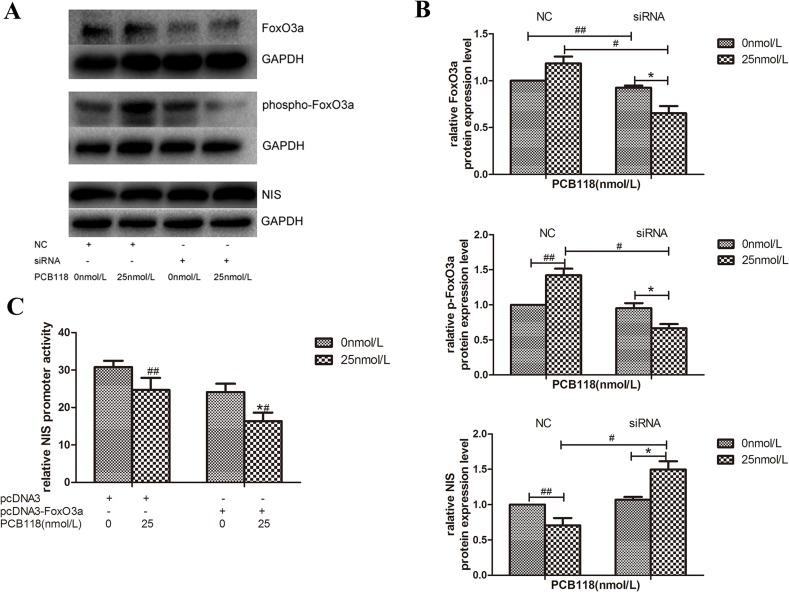
FoxO3a plays a role in the down-regulation of NIS expression in PCB118-treated FRTL-5 cells. FoxO3a was knocked down by FoxO3a-siRNA in FRTL-5 cells, and the cells were treated with PCB118 at 25 nM. Whole cell lysates were analyzed by western blotting using antibodies recognizing FoxO3a, p-FoxO3a, and NIS (A and B). FoxO3a over-expression was induced by the pcDNA3-FoxO3a plasmid, and the cells were harvested for the measurement of NIS promoter activity (C). Results show sections of blots from one experiment of the three that yielded similar results (A) or represent the mean ± SD of three independent experiments (B and C). GAPDH served as the loading control in western blotting.*p < 0.05, compared with the DMSO control group transfected with siRNA or the pcDNA3-FoxO3a plasmid; ^#^p < 0.05, compared with the 25 nM PCB118-treated group transfected with the NC or pcDNA3 plasmid; ^##^p < 0.05, compared with the DMSO control group transfected with the NC or pcDNA3 plasmid. The relative ratio of target protein/GAPDH and promoter activity in solvent control group was set as 1.

In our study, we found that the p-FoxO3a protein expression level increased (Fig. 4A, B) and FoxO3a promoter activity was enhanced after PCB118 treatment (Fig. 4D). Thus, we hypothesized that FoxO3a may play a role in regulating NIS expression. To examine whether FoxO3a regulated NIS expression, we designed siRNAs targeted to FoxO3a. Western blotting revealed that FoxO3a was efficiently downregulated by targeted siRNA in both the DMSO control group and 25 nM PCB118-treated groups (p < 0.05; Fig. 5A, B). The downregulation of FoxO3a resulted in decreased FoxO3a phosphorylation on stimulation with PCB118; thus, the protein expression levels of p-FoxO3a were also decreased by siRNA in the PCB118-treated group (p < 0.05; Fig. 5A and 5B). Compared with the PCB118-treated NC group and the DMSO control group, siRNA-induced FoxO3a downregulation led to an increase in NIS protein levels in the 25 nM PCB118-treated group (p < 0.05; Fig. 5A and B). To further study whether FoxO3a regulated NIS expression, the pcDNA3-FoxO3a or BC plasmid was co-transfected with the NIS promoter plasmid into FRTL-5 cells. As shown in Fig. 5C, after 48 h, FoxO3a over-expression led to significant reduction in NIS promoter activity (p < 0.05) in PCB118-treated group when compared to the BC group or DMSO control group (p < 0.05). These results may indicate that FoxO3a plays an important role in the down-regulation of NIS expression in PCB118-treated FRTL-5 cells.

### PCB118-induced reduction in NIS expression depends on the activation of the PI3K/Akt/FoxO3a signaling pathway

**Fig 6:**
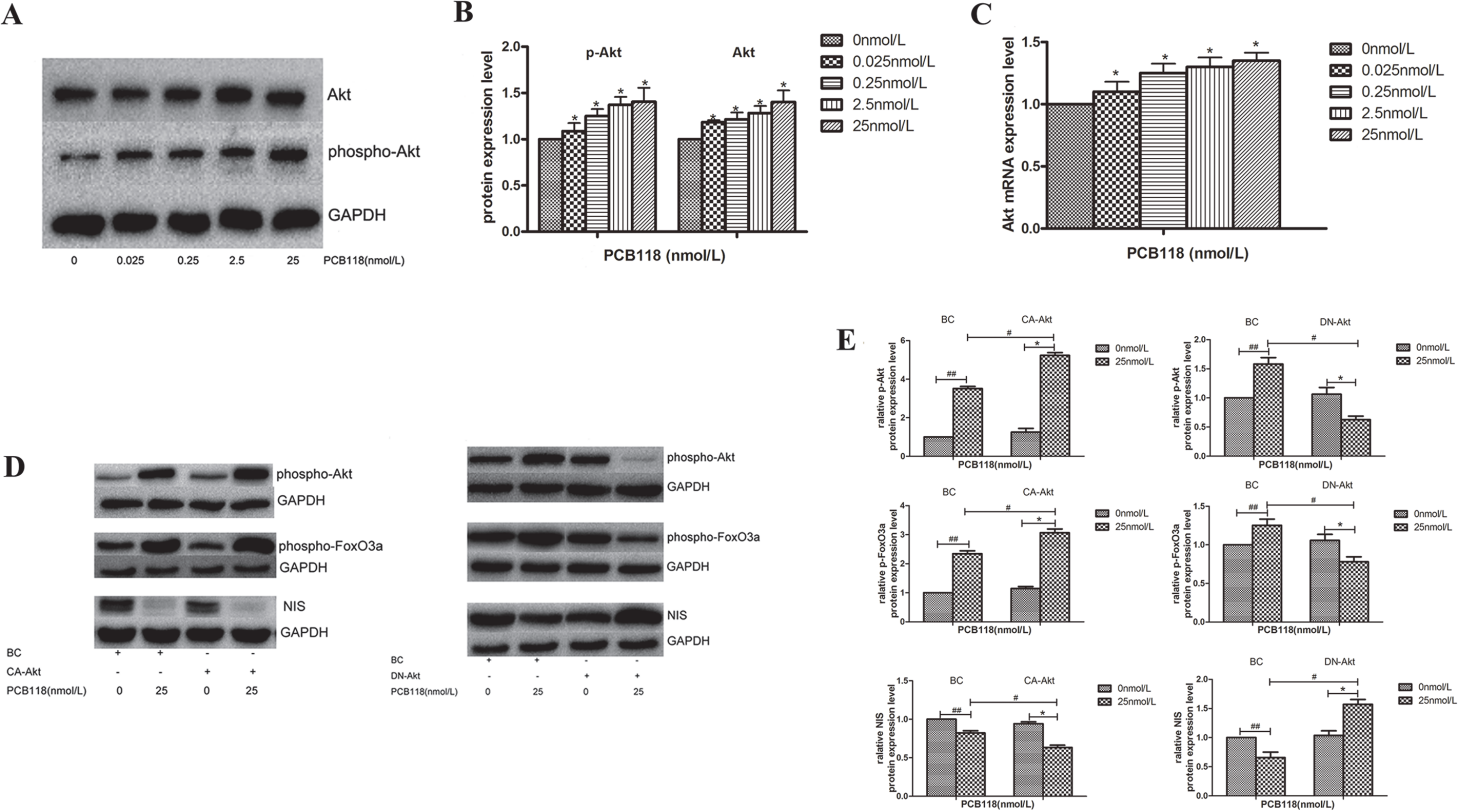
Akt pathway was activated in PCB118-treated FRTL-5 cells. Cells were treated with DMSO or various concentrations of PCB118 (0.025–25 nM) for 24 or 48 h. Whole cell lysates were analyzed by western blotting using antibodies recognizing Akt and p-Akt (A and B). Total RNA was isolated for qRT-PCR of Akt (C). Up-regulation of Akt expression was induced by CA-Akt and down-regulation was induced by DN-Akt in FRTL-5 cells. After treatment with 25 nM PCB118 for 48 h, whole cell lysates were analyzed by western blotting using antibodies recognizing p-Akt, p-FoxO3a, and NIS (D and E). Results show sections of blots from one experiment of the three that yielded similar results (A and D) or represent the mean ± SD of three independent experiments (B, C, and E). GAPDH served as the loading control in western blotting. *p < 0.05, compared with the DMSO control group (Fig. 6B, C) *p < 0.05, compared with the DMSO control group transfected with the CA-Akt or DN-Akt plasmid (Fig. 6E); ^#^p < 0.05, compared with the 25 nM PCB118-treated group transfected with the BC plasmid; ^##^p < 0.05, compared with the DMSO control group transfected with the BC plasmid. The relative ratio of target mRNA/β-actin and target protein/GAPDH in solvent control group was set as 1.

Fig. 6 shows that Akt and p-Akt protein levels and Akt mRNA levels increased significantly in the PCB118-treated groups compared with the DMSO control group (p < 0.05; Fig. 6 A, B, C). This result showed that the PI3K/Akt signaling pathway was activated in FRTL-5 cells after exposure to PCB118. To verify whether the activation of the PI3K/Akt signaling pathway could down-regulate NIS expression, the CA-Akt or DN-Akt plasmid was transfected into FRTL-5 cells. The cells were harvested for western blotting, and the variation in the p-Akt, p-FoxO3a, and NIS protein levels was examined. Compared to the DMSO control group and PCB118-treated BC (blank plasmid control) group respectively, the expression level of p-Akt and p-FoxO3a increased significantly and NIS expression decreased dramatically in 25nM PCB118-treated group when transfected with CA-Akt plasmid (p < 0.05), whereas the opposite results were obtained in PCB118-treated group transfected with DN-Akt plasmid (p < 0.05; Fig. 6D, E). Therefore, we suggest that PCB118 down-regulates NIS expression, perhaps through the activation of the PI3K/Akt/FoxO3a signaling pathway.

## Discussion

In a previous study, we demonstrated that persistent exposure to low PCB118 concentrations could severely damage thyroidal structure, dramatically decreasing the concentration of serum thyroid hormones. Furthermore, pivotal gene expression such as NIS and thyroglobulin, which determine the synthesis and secretion of thyroid hormones, was remarkably decreased in Wistar rats [6]. In the present study, we continued exploring the molecular mechanisms of FRTL-5 dysfunction related to low concentrations of PCB118 exposure in vitro.

PCBs consist of a series of 209 individual congeners according to the number and position of the substituent chlorine on a biphenyl structure and can be classified into three types of inducers: 2, 3, 7, 8-tetrachlorodibenzo-*p*-dioxin (TCDD)-type, phenobarbital (PB)-type, and mixed (TCDD/PB)-type inducers. 3,3′,4,4′,5-Pentachlorobiphenyl (PCB126) and 2,2′,4,4′,5,5′-hexachlorobiphenyls (PCB153) are representatives of the TCDD- and PB-type inducers, respectively [34]. In contrast, PCB118 is considered a mixed (TCDD/PB)-type inducer [34] (McFarland and Clarke 1989) and shares the properties of both TCDD- and PB-type inducers. In addition, PCB118 is one of the most persistent PCB congeners and has been found in human tissues and detected in human breast milk [35]. It is also one of the nine PCB congeners most strongly linked to thyroid dysfunction [36], and its contamination is widespread in water and soil and among the aquatic organisms of the Yangtze River Delta region of China. We selected PCB118 as the representative contaminant for the study.

In our previous in vivo study, we found significant deterioration of the thyroid structure and function even at a minimal dosage of 10μg/kg/day (approximately 6×10^4^ nM per day)[6], and previous study indicated that serum concentration of PCBs reach approximately 3×10^3^ nM in people exposed to PCBs [37]. These concentrations were out of the range of concentrations used in the in vitro experiment; however, the in vivo and in vitro experiments are different; thus, we referred to PCB concentrations from other in vitro studies [38,39]. Moreover, the concentration of our PCB118 stock solution was 25 mM to facilitate convenient dilutions into different concentrations, and we adopted concentrations of 0.025–25,000 nM to perform the CCK8 assay.

In the first step of the CCK-8 cell viability test, it was found that PCB118 inhibited cell viability in a concentration- and time-dependent manner at concentrations ranging from 0.025 nM to 25000 nM. Significant deterioration of cell viability was revealed at relatively high concentrations (250–25000 nM). According to this result, we selected the concentration range of 0.025–25 nM as the low PCB118 concentrations in subsequent research. It was found that when PCB118 was used at concentrations of 0.025–25 nM, it did not influence either cell viability or cell apoptosis. However, series variations in genes and proteins were observed, such as NIS, Akt, p-Akt, and p-FoxO3a. This may indicate that the damage caused by low concentrations of PCB118 to thyroid-specific genes was more remarkable than the influence of PCB118 on FRTL-5 cell viability.

On the other hand, such low PCB118 concentrations (0.025–25 nM) was far below the concentrations used in some other previous studies related to the effect of PCB exposure on thyroid and neuroendocrine systems [40–42]. However, these concentrations in our study were very similar to those of 1 pM to 1μM used by Rankouhi et al. in primary hepatocytes of fish, which had little or no influence on cell viability [43]. The difference in these detections may be caused by different types of PCBs, the duration of contaminant stimulation, or distinct characteristics of cells in each experiment, and the observed endpoints of respective studies.

Our existing results also found that at 0.025–25 nM of PCB118 could significantly inhibit both mRNA and protein expression levels of NIS, and NIS promoter activity was suppressed; this suggested that chronic PCB118 exposure posed a significant risk for thyroid dysfunction. The above mentioned findings are remarkably consistent with those of a previous study in which NIS gene activity was significantly down-regulated at 306 nM PCB126 [44] and our previous research *in vivo* that PCB118 could result in progressively lower FT3, FT4 and TSH concentrations in serum, led to histopathological deterioration of the thyroid (such as follicular hyperplasia and expansion, shedding of epithelial cells and fibrinoid necrosis), and cause significant decreases in NIS mRNA expression levels [6]. Other series of studies also indicated that PCBs could inhibit the process of thyroid hormone production [8,45], which may result from the reduction in NIS expression, yet the specific mechanism is unknown. It has been reported that TSH can down-regulate NIS expression through the Gβγ/PI3K/Akt pathway [46]. Moreover, Kogai et al. illustrated that the activation of the PI3K signaling pathway in TSH-stimulated thyroid cells causes a reduction in NIS expression at the transcription level [47]. It was also found that inhibition of the PI3K/Akt signaling pathway increased the expression of NIS mRNA and protein [48], as well as up-regulated the activation of the NIS promoter [49]. In our study, constitutive activation of Akt enhanced the decrease in PCB118-induced NIS expression level, whereas dominant-negative Akt was sufficient to reverse the decrease in PCB118-induced NIS expression level. These results suggest that the PI3K/Akt pathway was activated in PCB118-treated FRTL-5 cells. It is well established that the PI3K/Akt pathway is crucial for cellular processes [50]. Following its phosphorylation, Akt activates a number of downstream targets. However, as one of the most important downstream targets the PI3K/Akt pathway [18], whether FoxO3a plays a key role in regulating NIS expression is still unknown. In our study, in PCB118-treated FRTL-5 cells, the PI3K/Akt pathway was activated, and FoxO3a was phosphorylated by Akt, leading to its translocation to the cytoplasm and an elevation of p-FoxO3a. This signifies that at low PCB concentrations, total FoxO3a did not appear to significantly change, whereas the proportion of p-FoxO3a increased. Meanwhile, we also found the increased p-FoxO3a expression level could be reversed by FoxO3a-siRNA, simultaneously, accompanied with the increased NIS expression level. Moreover, the NIS promoter activity was notably reduced when overexpressing of FoxO3a. The aforementioned results suggested that FoxO3a may act as a transcription factor that regulates NIS expression.

In the study, we discovered that PCB118 down-regulates NIS expression in FRTL-5 cells, at least in part, through activated of PI3K/Akt pathway and enhanced p-FoxO3a which revealed the inactivated FoxO3a increased. Hence, our study indicates that the molecular mechanism of dysfunction of FRTL-5 thyrocyte cells induced by PCB118 involves the PI3K/Akt/FoxO3a pathway. This finding has advanced our understanding of the role of PCB118 in thyrocytes and provides a new concept that may prove effective in blocking the damage caused by PCBs to the human body. However, the role of the DNA binding site of FoxO3a in or around the NIS promoter area remains unclear. This is the goal of our further research using the electrophoretic mobility shift assay and chromatin immunoprecipitation.
